# Comparison of feature tracking with harmonic phase imaging analysis for the assessment of diastolic dysfunction

**DOI:** 10.1186/1532-429X-15-S1-E110

**Published:** 2013-01-30

**Authors:** Jonas Doerner, Daniel Kuetting, Alois M Sprinkart, Claas P Naehle, Hans H Schild, Daniel Thomas

**Affiliations:** 1 Radiology, University of Bonn, Bonn, Germany

## Background

Diastolic dysfunction (DD) is common in patients with cardiovascular disease. Current cardiac magnetic resonance techniques for assessment of DD require the acquisition of tagged imaging sequences, and complex post-processing. A new post-processing software allows for rapid strain assessment using feature tracking analysis (FT) (TomTec, Diogenes Software, Germany) based on conventional steady-state free precession (SSFP)-Cine sequences. The aim of this study was to compare tagging (TAG) with FT for assessment of diastolic function in patients with DD and healthy controls.

## Methods

20 healthy volunteers and 10 patients with echocardiographic diagnosed DD Grade II-III were investigated by the 2 techniques. Cardiovascular magnetic resonance imaging (Philips Intera 1.5T) using CSPAMM and SSFP-Cine sequences was performed for matched mid-ventricular short-axis slices. Each modality was analyzed offline using dedicated post-processing software (Tag Track, Gyrotools, CH for CSPAMM and TomTec for SSFP-Cine) and early-diastolic strain rate (EDSr) was calculated from both datasets. EDSr derived from CSPAMM and SSFP-Cine data were compared and inter-observer and intra-observer variability assessed using Bland-Altman analysis and Pearson correlation coefficient.

## Results

For all measurements FT EDSr (78.15+/- 17.17 s^-1^) was highly correlated with TAG EDSr (76.71+/- 17.45s^-1^), Bland-Altman analyses for method comparison yielded a mean difference of 1.4 (95% CI: 0.0 to 2.9). Limits of agreement (+/- 1.96 SD) were -14.9 and 12.0. In patients with DD FT EDSr (61.40+/- 15.60s^-1^) was not significantly different to TAG EDSr (57.60+/- 12.30s^-1^) (p=ns). The same was found in the control group FT EDSr (86.52+/- 10.55s^-1^) and TAG (86.27+/- 10.31s^-1^) (p=ns). Intra-observer variability yielded a mean difference of 1.2+/- 4.9 (FT) and 0.9+/- 3.5 (TAG). Inter-observer variability displayed a mean difference of 2.8+/- 5.84 (FT) and 0.3+/- 3.7 (TAG). Pearson correlation coefficient was (0.96 (FT); 0.97 (TAG)) for intra-observer variability and (0.94 (FT); 0.97 (TAG)) for inter-observer variability.

## Conclusions

Assessment of EDSr using a novel feature tracking technique shows good agreement with tagging derived data and may thus be used for rapid clinical determination of diastolic dysfunction. However, compared to feature tracking, tagging revealed lower intra - and inter-observer variability and may therefore remain the preferred technique for research applications.

## Funding

None.

**Figure 1:**
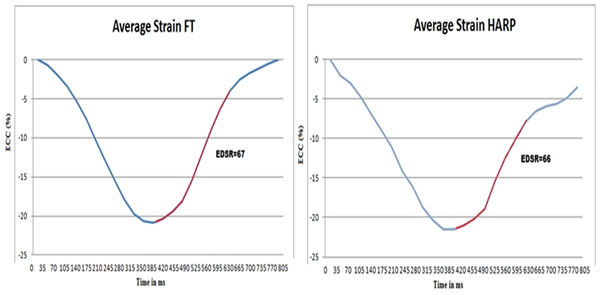
Representative myocardial strain analysis Utilizing both techniques (FT left side, TAG right side), diastolic strain (blue line) and EDSr (red line) was assessed in the same patient with diastolic dysfunction.

